# The Eye That Felt Alive: A Rare Case of Subconjunctival Loa loa Infection in the United Kingdom

**DOI:** 10.7759/cureus.105082

**Published:** 2026-03-12

**Authors:** Awni Aburamadan, Noor Sadiq Syed, Aboubakr Mohamed, Akaninyene Otu, Stuart Bond, Andrew Chung, Mahmoud Eltawagny

**Affiliations:** 1 Ophthalmology, Mid Yorkshire Teaching NHS Trust, Wakefield, GBR; 2 Emergency Medicine, Mid Yorkshire Teaching NHS Trust, Wakefield, GBR; 3 Microbiology, Mid Yorkshire Teaching NHS Trust, Wakefield, GBR; 4 Pharmacy, Mid Yorkshire Teaching NHS Trust, Wakefield, GBR; 5 Emergency, King's Mill Hospital, Sutton-in-Ashfield, GBR

**Keywords:** emergency eye management, medically important parasites, ocular parasites, ophthalmology, rural emergency medicine

## Abstract

We present the case of a 28-year-old Nigerian woman who presented to the Emergency Department with the sensation that “something was moving” in her right eye. Examination revealed a live, mobile parasite migrating beneath the bulbar conjunctiva near the limbus. Laboratory investigations demonstrated eosinophilia. Urgent surgical extraction was performed, and morphological analysis confirmed *Loa loa*.

This case highlights the importance of recognising tropical parasitic infections in non-endemic regions, the value of multidisciplinary management, and considerations for systemic therapy, particularly in pregnancy.

## Introduction

*Loa loa*, commonly referred to as the African eye worm, is a filarial nematode endemic to the forested regions of West and Central Africa and transmitted to humans through the bite of infected *Chrysops* (deer fly) species [1]. Infection may lead to a spectrum of clinical manifestations, ranging from asymptomatic microfilaremia to transient subcutaneous swellings known as Calabar swellings and migration of adult worms through subcutaneous tissues.

One of the most characteristic manifestations of loiasis is the migration of an adult worm across the subconjunctival space, producing the classic “eye worm” phenomenon, which may cause ocular irritation, foreign body sensation, and patient distress [1,2]. Although such presentations are well recognised in endemic regions, they are rarely encountered in non-endemic countries.

With increasing global migration and international travel, sporadic cases are now being reported in Europe and other non-endemic regions, sometimes many years after initial exposure [3,4]. Awareness of this condition is therefore important for clinicians practising in non-endemic settings, as early recognition and appropriate multidisciplinary management are essential for accurate diagnosis and safe treatment.

## Case presentation

A Nigerian woman in her 20s, who had migrated to the United Kingdom four years earlier, presented to the Emergency Department with an acute sensation of movement in her right eye. She reported observing a slender, motile structure beneath the conjunctiva while looking in a mirror. Symptoms were limited to mild soreness without visual disturbance.

She reported living in Nigeria prior to migrating to the United Kingdom four years earlier and had not returned to endemic regions since that time. She did not recall previous episodes suggestive of Calabar swellings or other systemic manifestations of loiasis.

**Figure 1:**
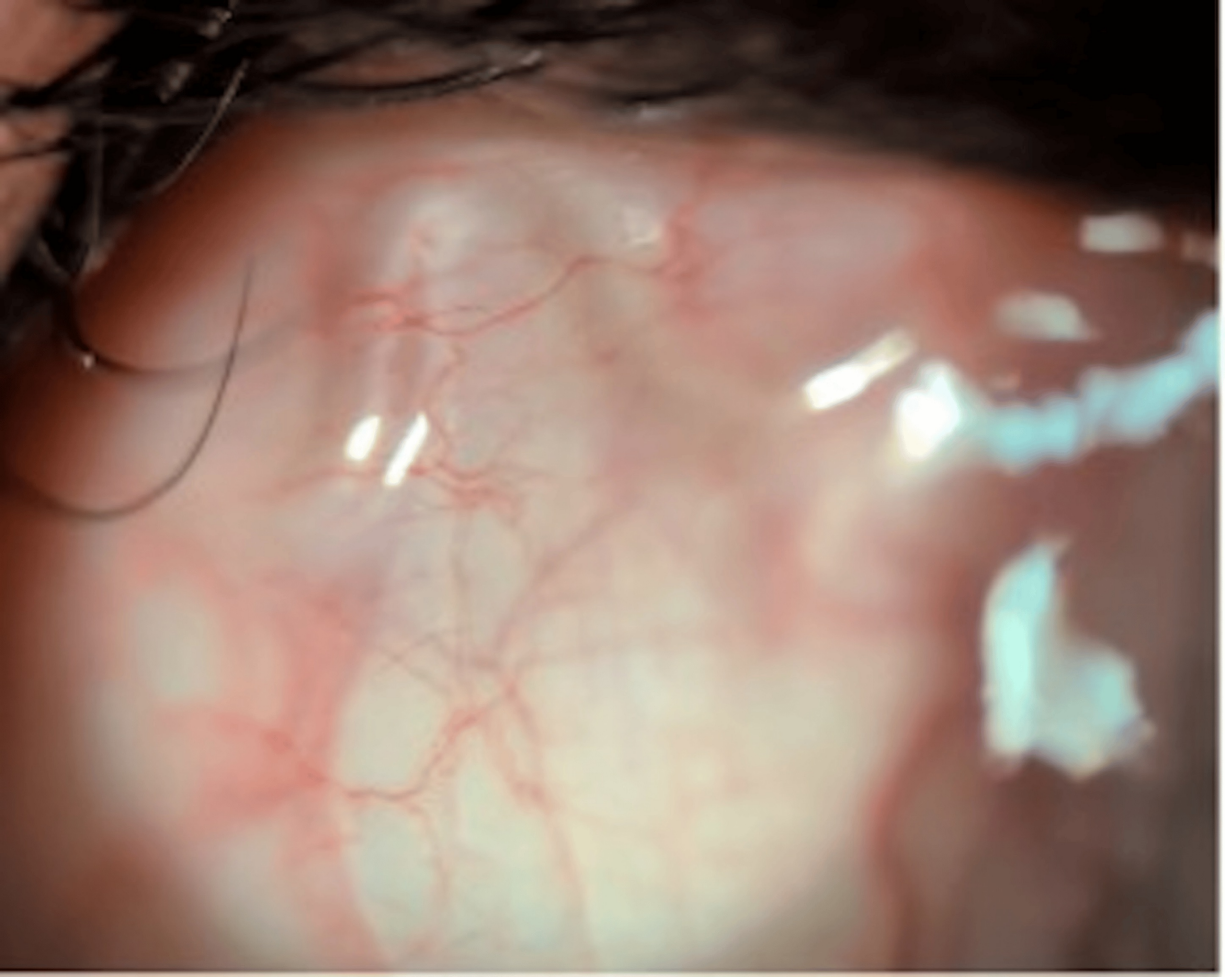
Slit-lamp photograph demonstrating a motile, translucent worm beneath the bulbar conjunctiva near the limbus.

Visual acuity was 6/6 bilaterally. Slit-lamp examination revealed a live, translucent, thread-like worm measuring approximately 28-30 mm, migrating beneath the bulbar conjunctiva near the lateral limbus. This finding is demonstrated in Figure 1. The cornea, anterior chamber, vitreous, and retina were normal.

Laboratory investigations demonstrated eosinophilia (1.09 × 10⁹/L) and an elevated erythrocyte sedimentation rate (46 mm/hr), consistent with parasitic infection [5]. Renal and liver function tests were normal. Filarial serology was strongly positive.

**Figure 2:**
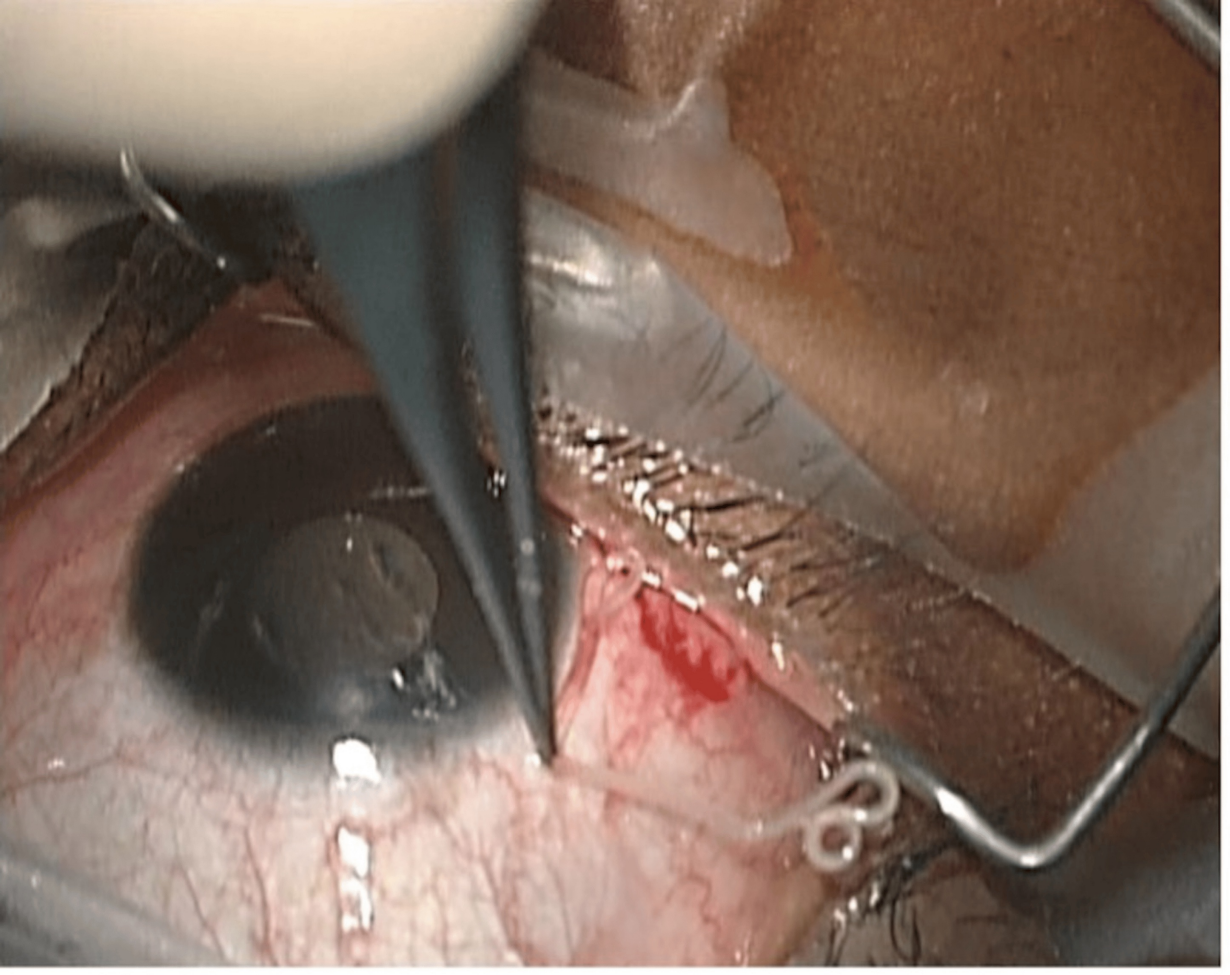
Microscopic laboratory image demonstrating the extracted adult male Loa loa worm.

**Figure 3:**
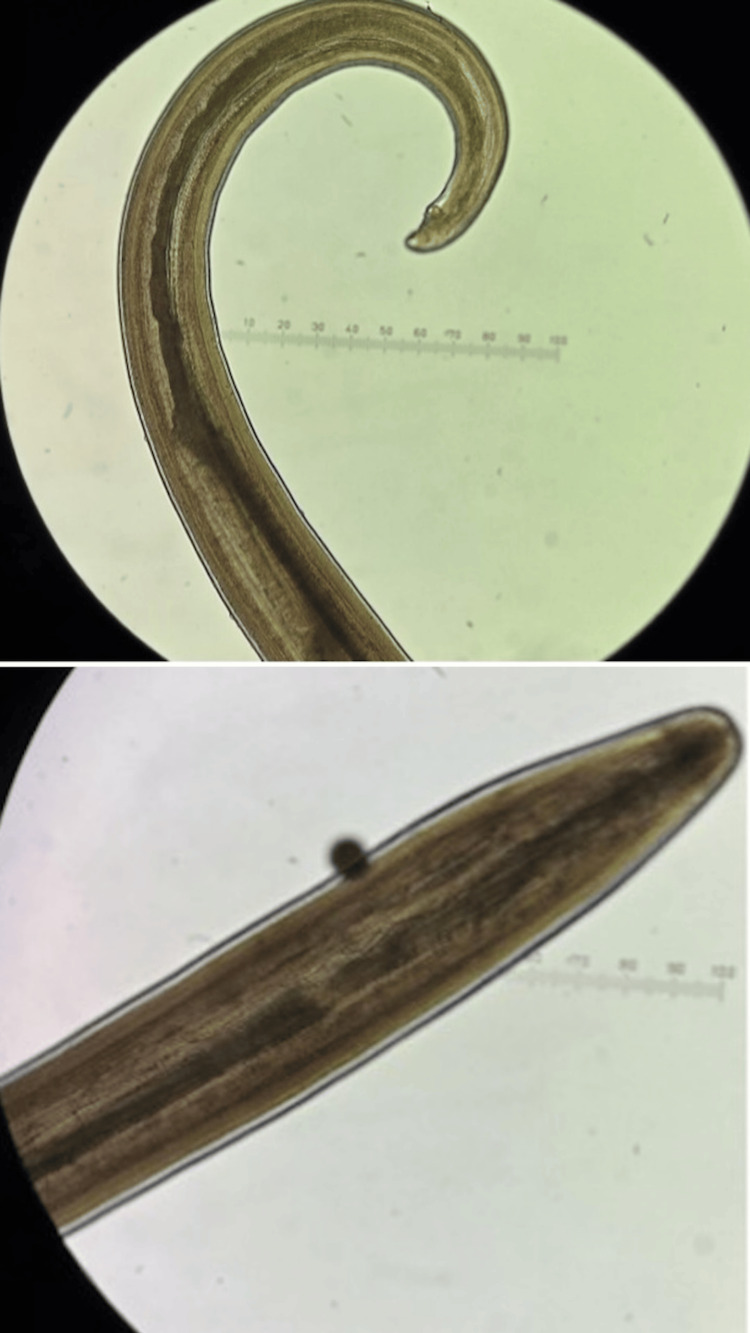
Laboratory microscopic photo identifying male Loa loa worm.

Urgent surgical extraction was performed under topical anaesthesia. A small subconjunctival peritomy was created, and the intact, motile worm was removed without complication. The intraoperative removal of the parasite is shown in Figure 2. The specimen was sent to the Hospital for Tropical Diseases, London, where morphological analysis confirmed a male *L. loa* worm. Microscopic identification of the extracted parasite is illustrated in Figure 3.

Postoperatively, topical chloramphenicol and prednisolone acetate were prescribed. At the one-week review, the conjunctiva was healing without recurrence.

Daytime blood sampling between 10:00 and 14:00, in keeping with the known diurnal periodicity of *L. loa* microfilariae [1], did not demonstrate circulating microfilariae. Although concentration techniques or repeat testing may improve detection sensitivity, further sampling was not pursued, as the diagnosis had already been confirmed through direct visualisation and surgical extraction of the adult worm. Shortly thereafter, the patient was confirmed to be approximately six weeks pregnant. Following multidisciplinary discussion with infectious diseases and tropical medicine specialists, systemic therapy with diethylcarbamazine (DEC) was deferred until postpartum due to limited safety data in pregnancy and the need for careful microfilarial load assessment prior to treatment [6].

## Discussion

This case illustrates the delayed presentation of ocular loiasis in a non-endemic region, several years after migration, reflecting the prolonged lifespan of adult worms [1]. Increasing international mobility has resulted in reported cases across Europe, including Switzerland and France [3,4].

The hallmark of ocular loiasis is direct visualisation of a motile, translucent worm beneath the conjunctiva [2]. Differential diagnoses for subconjunctival parasites include *Dirofilaria* species and other filarial infections. Epidemiological history, combined with morphological identification, confirms the diagnosis. Other rare causes of subconjunctival parasitic infection include *Thelazia* species and other filarial nematodes, although these are considerably less common and are typically associated with different epidemiological exposures.

Laboratory findings commonly include eosinophilia and elevated inflammatory markers [5]. Microfilaremia demonstrates diurnal periodicity, with peak detection between 10:00 and 14:00 [1]. Absence of detectable microfilariae does not exclude infection.

Surgical extraction is the primary intervention in ocular involvement, providing immediate symptomatic relief and enabling species identification [2,7]. Postoperative topical antibiotics and corticosteroids help reduce inflammation and the risk of secondary infection [3].

Systemic therapy aims to eradicate residual adult worms and circulating microfilariae. DEC is the first-line treatment for loiasis [6]. However, patients with high microfilarial loads are at risk of severe adverse reactions, including encephalopathy [6]. Co-infection with onchocerciasis must be excluded prior to DEC initiation, due to the risk of Mazzotti reaction [8]. Alternative agents, such as albendazole or ivermectin, may reduce microfilarial burden, although ivermectin is not curative for adult worms [8]. Assessment of microfilarial load prior to treatment is important, as high microfilarial densities have been associated with severe inflammatory reactions and encephalopathy following treatment.

In pregnancy, safety data for DEC remain limited, and treatment decisions must be individualised through specialist, multidisciplinary input [6].

## Conclusions

Ocular *L. loa* infection, though rare in the United Kingdom, should be considered in patients presenting with subconjunctival, motile parasites and relevant epidemiological exposure. Direct visualisation is diagnostic, and surgical extraction provides definitive management. Systemic therapy requires careful evaluation of microfilarial burden and coordination between ophthalmology, infectious diseases, and tropical medicine services, particularly in pregnancy.
